# Supplementary material for: DAF-2c signaling promotes taste avoidance after starvation in Caenorhabditis elegans by controlling distinct phospholipase C isozymes
Source: Commun Biol. 2022 Jan 11;5:30. doi: 10.1038/s42003-021-02956-8 (PMC8752840; doi:10.1038/s42003-021-02956-8)
Supplement: Supplementary file 2 — Supplementary Information [file 42003_2021_2956_MOESM2_ESM.pdf]

Supplementary Fig 1

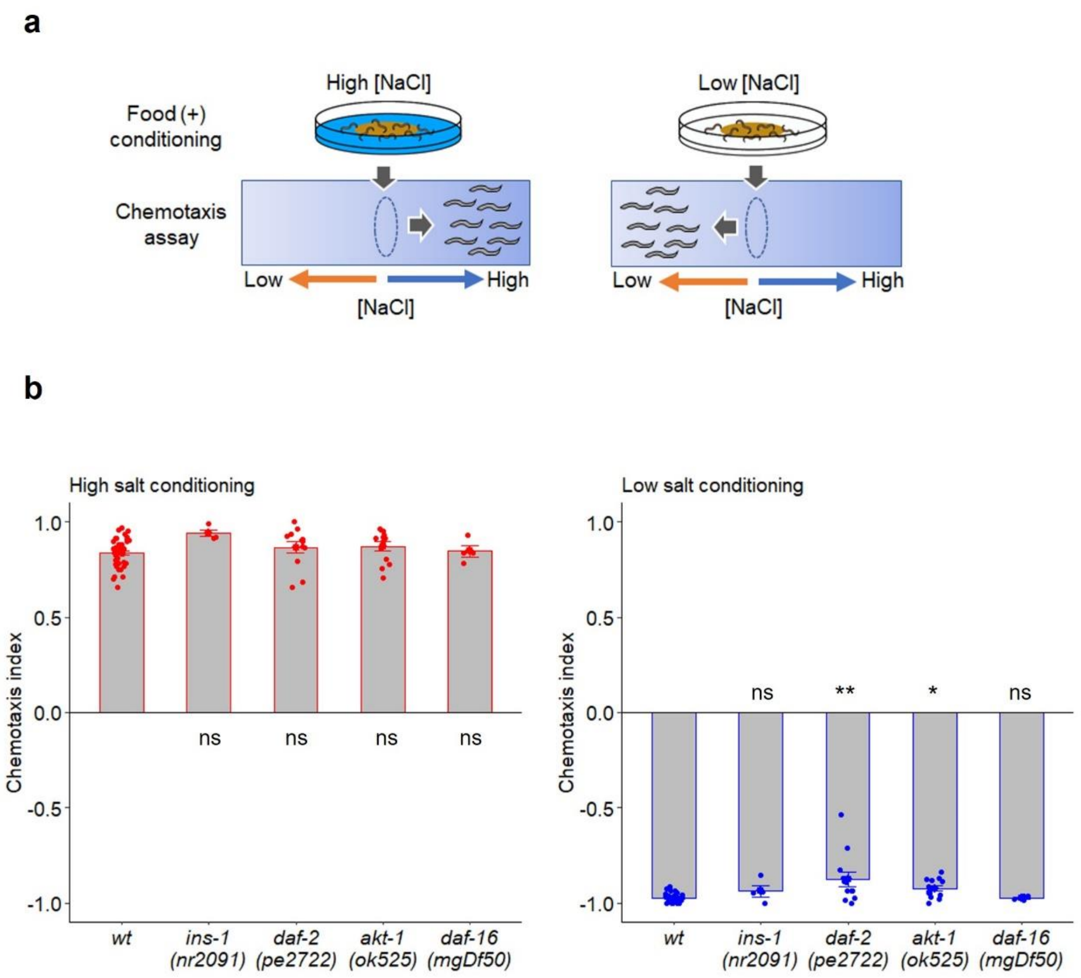

Supplementary Fig 1. Salt chemotaxis in insulin-like-pathway mutants after conditioning with feeding.

(a) Schematic of salt chemotaxis based on salt concentration memory. Worms are attracted to the salt concentrations that they encountered during feeding. (b) Salt chemotaxis after conditioning on agar plates at high or low salt concentrations in the presence of food. Each dot in red or blue represents a chemotaxis index obtained in each chemotaxis assay after conditioning at high or low salt concentrations, respectively. n = 4-36 assays. Bars represent mean values; error bars represent SEM. ANOVA with Dunnett's post hoc test: $*P < 0.05$ and $**P < 0.01$ compared to wt.

Supplementary Fig 2

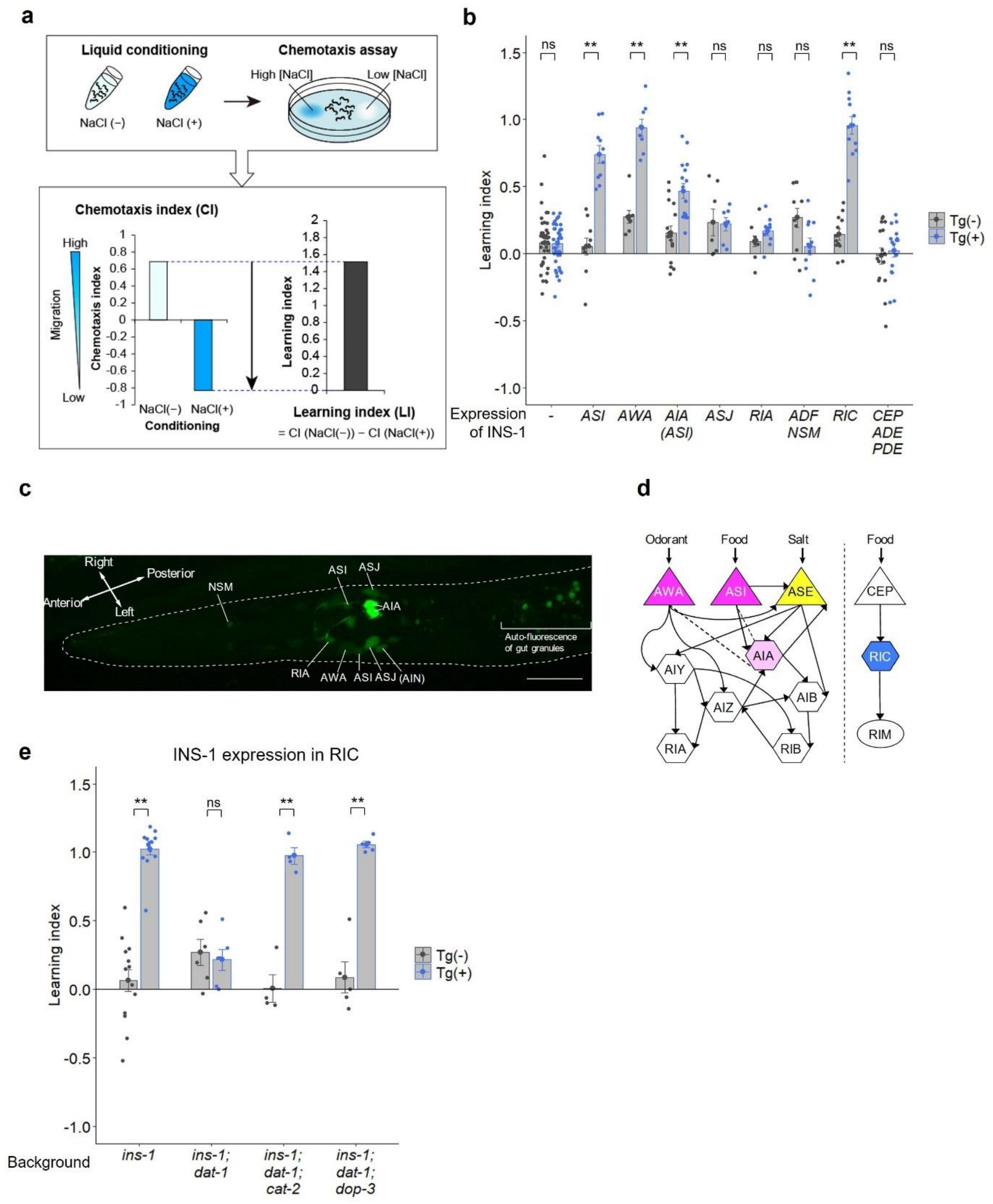

Supplementary Fig 2. The site of action of INS-1 in taste avoidance learning.

(a) Method for calculating the learning index: see details in Materials and Methods. (b) Effects of INS-1 expression in a single class of neurons or a subset of neurons on learning index. Salt chemotaxis assays were performed after liquid conditioning using *ins-1* mutant worms with or without transgenes for INS-1::Venus expression under the cell-type-specific

promoters; the learning indices was then calculated. n = 7-40 assays. (c) Expression pattern of an *ins-1* fosmid reporter. A z-stack confocal image of the head region of a larva is shown. Scale bar = 20 µm. (d) Schematic of a neural circuit downstream of AWA, ASI, ASE, and CEP sensory neurons. (e) Effects of INS-1 expression in RIC on learning index in the indicated genetic backgrounds. n = 4-14 assays. Each dot in blue or black represents a value calculated in each chemotaxis learning assay using worms with or without transgenes, respectively. Bars represent mean values; error bars represent SEM. Two-tailed Welch's t-test with Holm correction: $*P < 0.05$ and $**P < 0.01$.

Supplementary Fig 3

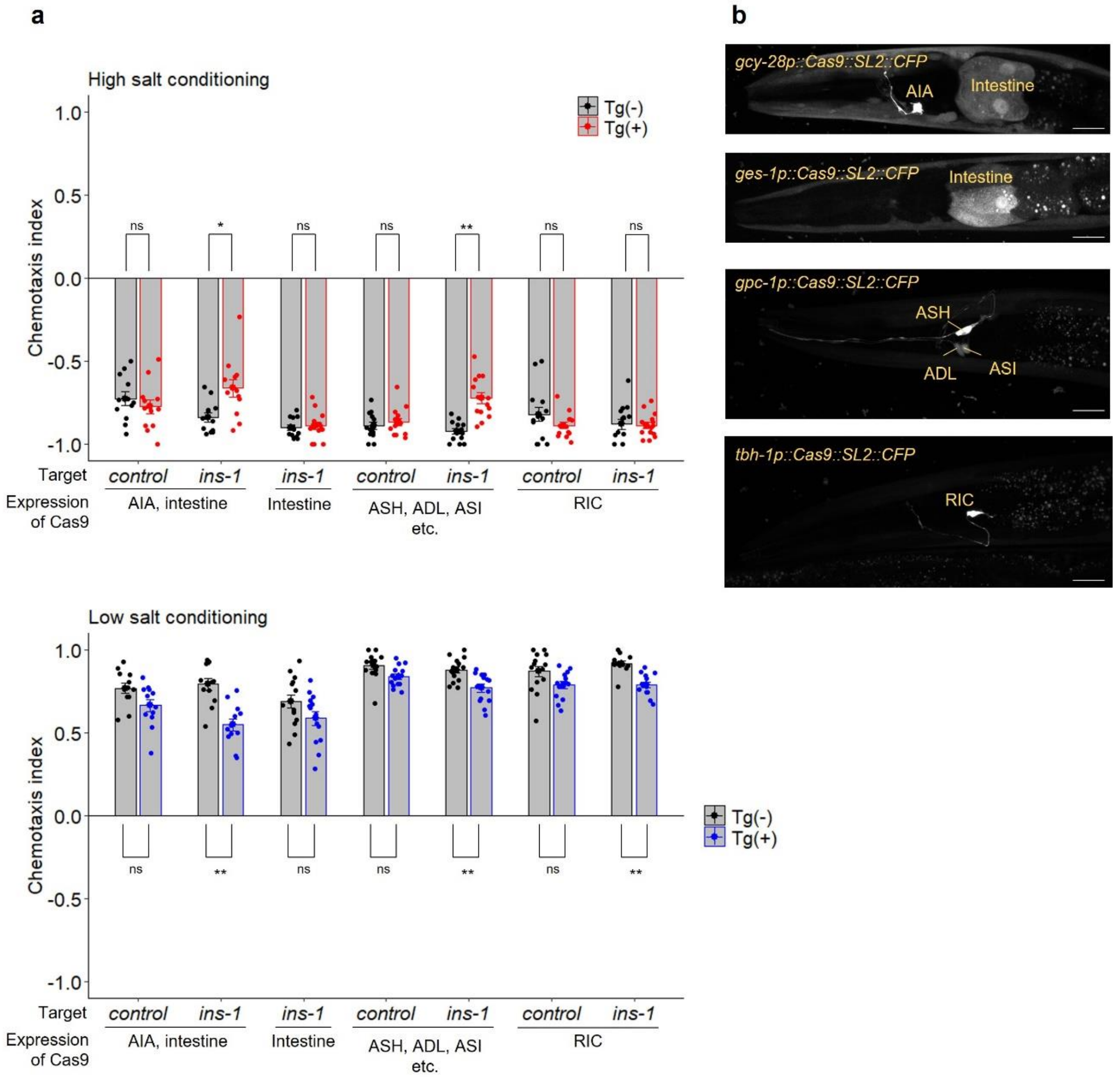

Supplementary Fig 3. Effects of cell-type-specific knockdown of *ins-1* on taste avoidance learning.

(a) Salt chemotaxis assays were performed after salt conditioning in the absence of food using worms expressing Cas9 in the intestine and AIA neurons under the *gcy-28d* promoter, in the intestine under the *ges-1* promoter, in chemosensory neurons including ASH, ADL and ASI under the *gpc-1* promoter or in RIC neurons under the *tbh-1* promoter and sgRNA with an *ins-1* target sequence under the *U6* promoter. sgRNA without a targeting sequence was used as a negative control. Each dot represents a chemotaxis index calculated in each chemotaxis assay using worms with (Tg(+)) or without (Tg(-)) transgenes. n = 12-14 assays. Two-tailed Welch's t-test with Holm correction: $*P < 0.05$ and $**P < 0.01$. (b) Confirmation of expression patterns of Cas9 fused with SL2::CFP in worms used for salt chemotaxis assay shown in (a). Scale bar = 20 µm.

Supplementary Fig 4

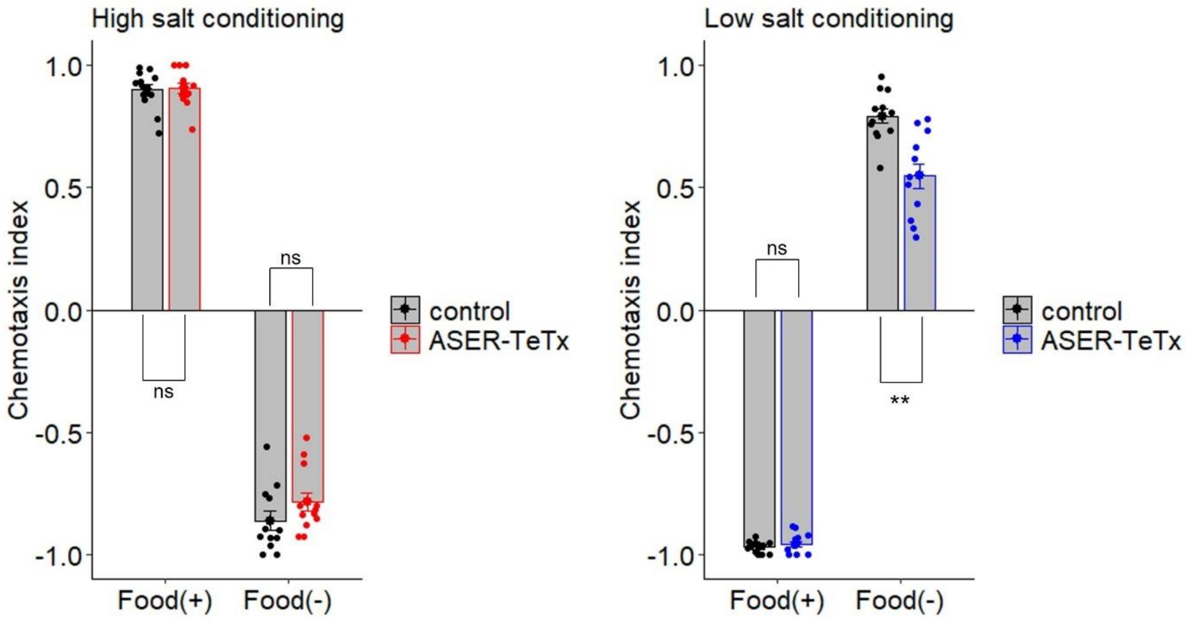

Supplementary Fig 4. Effects of TeTx expression in ASER on salt chemotaxis and taste avoidance learning.

Salt chemotaxis after conditioning on agar plates at high (left) or low (right) salt concentrations in the presence or absence of food using worms expressing TeTx in ASER. Each dot in red or blue represents a value calculated in each chemotaxis assay after conditioning at high or low salt concentrations, respectively. A black dot represents a chemotaxis index calculated in each chemotaxis assay using worms without transgenes. n = 12 assays. Bars represent mean values; error bars represent SEM. Two-tailed Welch's t-test with Holm correction: $^{**}P < 0.01$.

Supplementary Fig 5

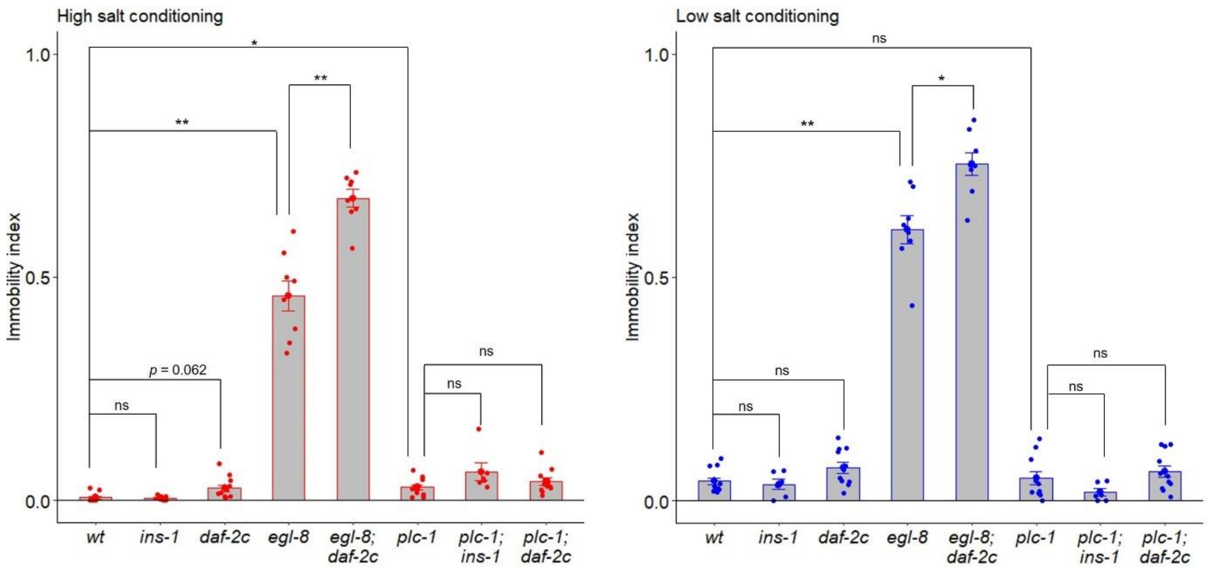

Supplementary Fig 5. *egl-8* mutants show low mobility in salt chemotaxis assay. Immobility on chemotaxis test plates after conditioning on agar plates at high (left) or low (right) salt concentrations in the absence of food. An immobility index represents ratios of worms that retained in the starting position after 45 min of salt chemotaxis test. An immobility index was determined according to the following equation: Immobility index = N_C / N_{all}, where N_{all} is the total number of worms on a test plate, and N_C is the number of worms in the area around the starting position. Each dot in red or blue represents a value calculated in each chemotaxis assay after conditioning at high or low salt concentrations, respectively. n = 6-17 assays. Bars represent mean values; error bars represent SEM. Two-tailed Welch's t-test with Holm correction: *$P < 0.05$ and **$P < 0.01$.

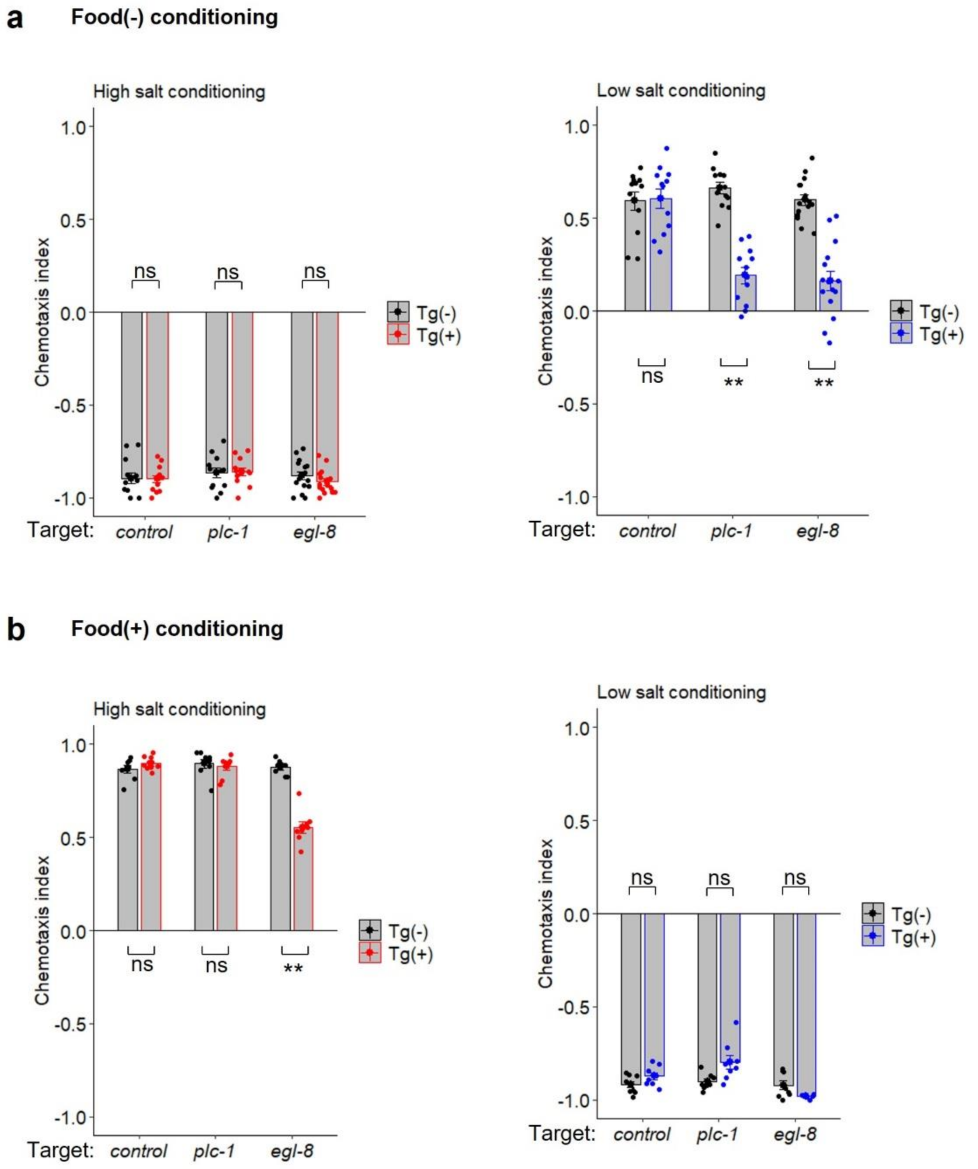

Supplementary Fig 6. Effects of ASER-knockdown of *plc-1* or *egl-8* on salt chemotaxis in the wild type.

Salt chemotaxis after conditioning on agar plates at high or low salt concentrations in the absence (a, n = 11-12) or presence (b, n = 8) of food using worms expressing Cas9 in ASER and sgRNA with a target sequence of *plc-1* or *egl-8*. sgRNA without a targeting sequence was used as a negative control. Each dot in red or blue represents the chemotaxis index calculated in each chemotaxis assay after conditioning at high (left) or low (right) salt concentrations, respectively. A black dot represents a chemotaxis index calculated in each chemotaxis assay using worms without transgenes. Two-tailed Welch's t-test with Holm correction: ***P* < 0.01.

Supplementary Fig 7

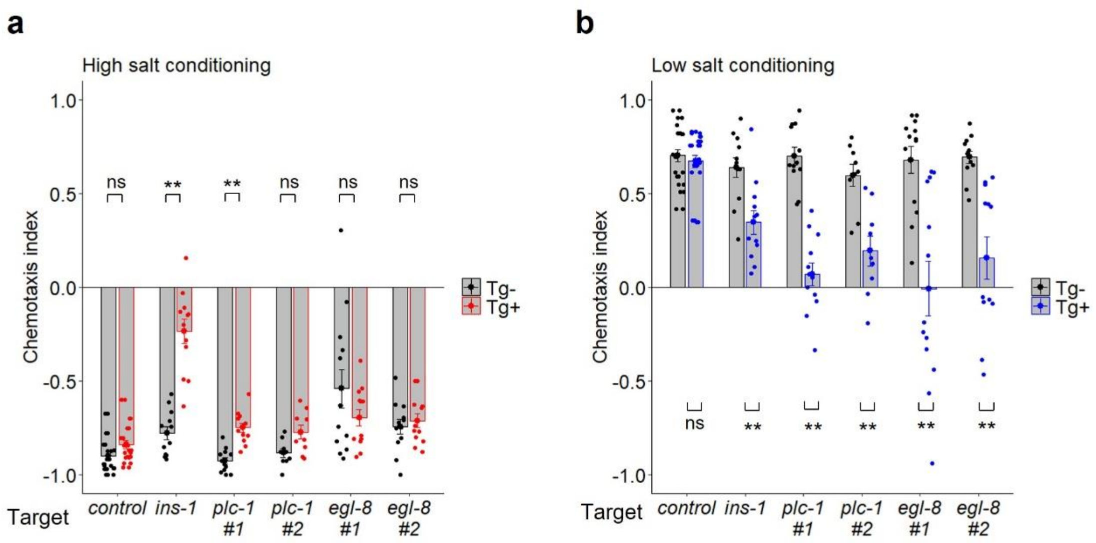

Supplementary Fig 7. Validation of targeting sequences for cell-specific knockdown using the somatic CRISPR/Cas9 strategy.

Effects of neuron-specific knockdown of *ins-1*, *plc-1*, and *egl-8* on taste avoidance learning. Salt chemotaxis assays were performed after salt conditioning in the absence of food using worms with or without transgenes for pan-neuronal Cas9 expression under the *rgef-1* promoter and sgRNA expression under the *U6* promoter. Each dot in red or blue represents a value calculated in each chemotaxis assay after conditioning at high (a) or low (b) salt concentrations, respectively. A black dot represents a chemotaxis index calculated in each chemotaxis assay using worms without transgenes. One or two (#1 and #2) targeting sequence(s) were examined for each gene, whereas sgRNA without a targeting sequence was used as a negative control. n = 9-21 assays. Two-tailed Welch's t-test with Holm correction: $^{**}P < 0.01$.
