# Supplementary material for: DAF-2c signaling promotes taste avoidance after starvation in Caenorhabditis elegans by controlling distinct phospholipase C isozymes
Source: Commun Biol. 2022 Jan 11;5:30. doi: 10.1038/s42003-021-02956-8 (PMC8752840; doi:10.1038/s42003-021-02956-8)
Supplement: Supplementary file 3 — Description of Additional Supplementary Files [file 42003_2021_2956_MOESM3_ESM.pdf]

Description of Additional Supplementary Data Files

File name: Supplementary Data 1
Description: List of mutant and transgenic *C. elegans* strains.
